# Autoantibody to MOG suggests two distinct clinical subtypes of NMOSD

**DOI:** 10.1007/s11427-015-4997-y

**Published:** 2016-02-26

**Authors:** Yaping Yan, Yujing Li, Ying Fu, Li Yang, Lei Su, Kaibin Shi, Minshu Li, Qiang Liu, Aimee Borazanci, Yaou Liu, Yong He, Jeffrey L. Bennett, Timothy L. Vollmer, Fu-Dong Shi

**Affiliations:** 1 Departments of Neurology and Immunology, Tianjin Neurological Institute, Tianjin Medical University General Hospital, Tianjin 300052, China; 2 Department of Neurology, Barrow Neurological Institute, St. Joseph’s Hospital and Medical Center, Phoenix 85013, USA; 3 State Key Laboratory of Cognitive Neuroscience and Learning and IDG/McGovern Institute for Brain Research, Beijing Normal University, Beijing 100875, China; 4 Department of Neurology, University of Colorado School of Medicine, Aurora 80045, USA

**Keywords:** MOG antibody, AQP4 antibody, neuromyelitis optica spectrum disorder, phenotype

## Abstract

We characterized a unique group of patients with neuromyelitis optica spectrum disorder (NMOSD) who carried autoantibodies of aquaporin-4 (AQP4) and myelin-oligodendrocyte glycoprotein (MOG). Among the 125 NMOSD patients, 10 (8.0%) were AQP4- and MOG-ab double positive, and 14 (11.2%) were MOG-ab single positive. The double-positive patients had a multiphase disease course with a high annual relapse rate (*P*=0.0431), and severe residual disability (*P*<0.0001). Of the double-positive patients, 70% had MS-like brain lesions, more severe edematous, multifocal regions on spinal magnetic resonance imaging (MRI), pronounced decreases of retinal nerve fiber layer thickness and atrophy of optic nerves. In contrast, patients with only MOG-ab had a higher ratio of monophasic disease course and mild residual disability. Spinal cord MRI illustrated multifocal cord lesions with mild edema, and brain MRIs showed more lesions around lateral ventricles. NMOSD patients carrying both autoantibodies to AQP4 and MOG existed and exhibited combined features of prototypic NMO and relapsing-remitting form of MS, whereas NMOSD with antibodies to MOG only exhibited an “intermediate” phenotype between NMOSD and MS. Our study suggests that antibodies against MOG might be pathogenic in NMOSD patients and that determination of anti-MOG antibodies maybe instructive for management of NMOSD patients.

## INTRODUCTION

Damage to astrocytes mediated by aquaporin-4 antibody (AQP4-ab) has been implicated as the cause of neuromyelitis optica spectrum disorder (NMOSD) (Lennon et al., 2005; Ratelade et al., 2012; Saadoun et al., 2010). Despite using the best available assays, 10%–30% of patients with NMO and NMOSD still test negative for AQP4-ab (Granieri et al., 2012; Papadopoulos and Verkman, 2012; Waters et al., 2012). This suggests that other autoantigens might exist in AQP4-ab negative patients and drive the disease progress. Current clinical practices, however, do not distinguish AQP4-ab-positive from -negative NMOSD patients. Nevertheless, published literature has suggested that these two groups of patients may have distinctive clinical features (Jarius et al., 2012; Jiao et al., 2014; Kitley et al., 2013). Importantly, we do not know whether both groups would respond similarly to disease modifying therapies. Thus, the identification of disease markers, including additional autoantibodies, and their roles in relevant pathophysiological processes would be instructive in diagnosis and management of these NMOSD patients.

Myelin antigens such as myelin oligodendrocyte glycoprotein (MOG) may not only be targeted secondarily in late stage of NMOSD, but also serve as autoantigens driving immune response in NMOSD, particularly in AQP4 antibody negative patients. The presence of antibodies against MOG revealed in several recent studies suggests that this could be the case (Kitley et al., 2014; Kitley et al., 2012; Mader et al., 2011; Rostasy et al., 2013; Sato et al., 2014; Tanaka and Tanaka, 2014). Although MOG autoantibodies (MOG-ab) were identified in NMOSD and later confirmed in six independent studies (Kitley et al., 2014; Kitley et al., 2012; Mader et al., 2011; Rostasy et al., 2013; Sato et al., 2014; Tanaka and Tanaka, 2014), whether NMOSD patients can carry antibodies to both AQP4 and MOG remains controversial (Kezuka et al., 2012; Kitley et al., 2014; Weinshenker and Wingerchuk, 2014; Woodhall et al., 2013). If so, do they exhibit any unique clinical characteristics? In this study, we used highly sensitive fluorescence-activated cell sorting (FACS) analysis methods (Reindl et al., 2013; Zhou et al., 2006) and identified AQP4-ab and MOG-ab dual positivity in a relatively large cohort from two sites. We further compared the clinical features, MRIs, and laboratory characteristics of double-positive NMOSD patients with their counterparts who were positive for only one of these autoantibody types.

## RESULTS

### Detection of serum MOG-ab and AQP4-ab in patients with NMOSD

**Figure 1:**
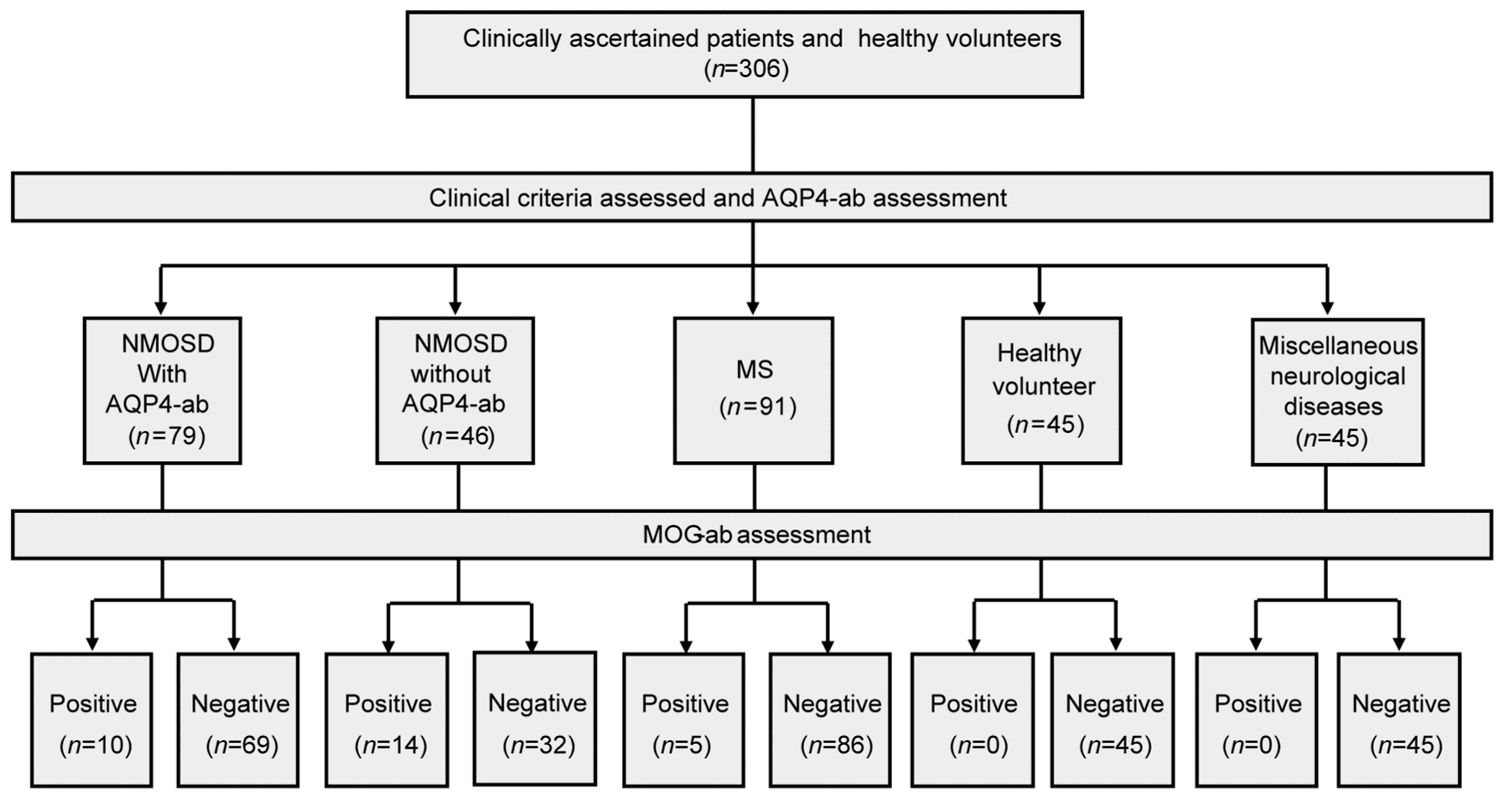
Study design for recruitment of patients for MOG-ab and AQP4-ab assessment.

All serums of eligible patients were consecutively collected from April to December 2013. FACS assay method was used for both full-length MOG-ab and AQP4-ab detection. Of the 125 NMOSD patients included in this study, 99 were from Tianjin Medical University General Hospital and 26 from Barrow Neurological Institute. The distributions of disease phenotypes were similar in the two cohorts (NMO: LETM:ON patients) for Tianjin and Barrow, 76:20:3 and, 21:4:1, respectively). As shown in Figure 1, sera from 125 NMOSD and 91 RRMS adult patients, all of whom were in remission status. The serum of 12/125 NMOSD patients were collected on no treatment (Table 1).

**Figure 2:**
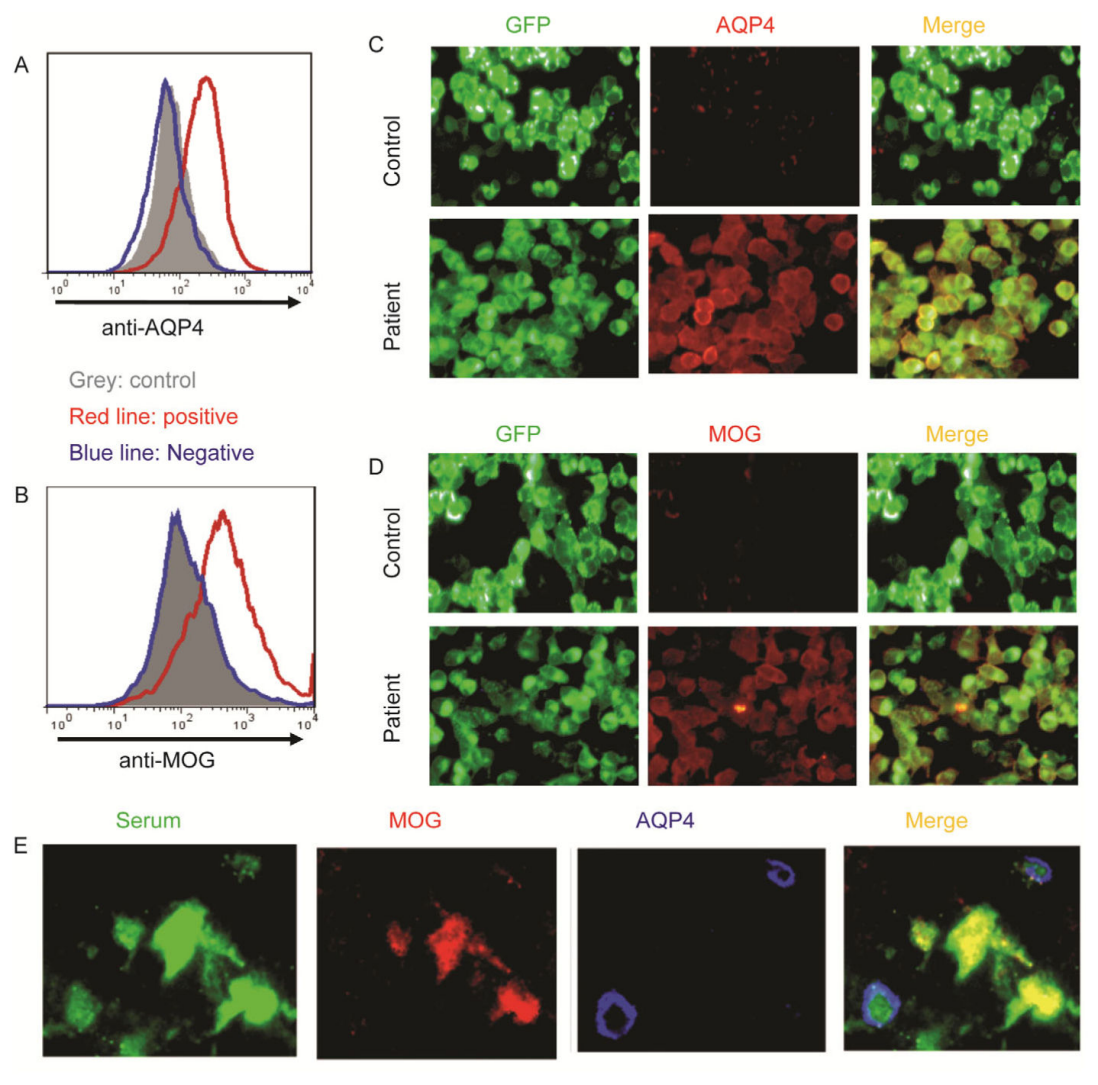
Representative detection of AQP4-ab and MOG-ab in a patient with NMOSD. A, AQP4-ab detection by the Fluorescence-activated cell sorting (FACS) analysis. B, MOG-ab detection by the Fluorescence-activated cell sorting (FACS) analysis. C, AQP4-ab detection by the fluorescence microscope-based cell binding assay. D, MOG-ab detection by the fluorescence microscope-based cell binding assay. E, The fixed sections of rat brain or spinal cord were co-stained with diluted patient serum (green), rabbit anti-human AQP4 (red) and goat anti-human MOG polyclonal antibodies (blue). In sections from the AQP4-ab and MOG-ab double-positive patients, merged green-red and merged green-blue are visible.

In total, 55.2% of the NMOSD patients (NMO:LETM: ON=57:8:4) were AQP4-ab single positive, and 11.2% of patients (NMO:LETM:ON=8:6:0) were MOG-ab single positive. Interestingly, 8% of our NMOSD patients were double positive for both MOG-ab and AQP4-ab (NMO: LETM:ON=9:1:0) (Figure 2); of these, seven were from Tianjin and three from Barrow. The remaining 25.6% of the patients (NMO:LETM:ON=23:9:0) were double negative. Five of 91 MS patients were positive for MOG-ab, but none of the MS patients were AQP4-ab positive or double positive (Figure 1).

### NMOSD patients with different autoantibody profiles exhibit distinct clinical features

To compare the clinical characteristics of our NMOSD patients, we arbitrarily divided them into three groups: patients with AQP4-ab but no MOG-ab (AQP4-ab positive), patients with MOG-ab but no AQP4-ab (MOG-ab positive) and patients with both antibody types (double positive). The clinical characteristics of each double-positive patient are summarized in Table 2.

Table 3 lists the demographic and clinical features of these patients. The median disease duration was similar in all three groups (AQP4-ab positive vs. MOG-ab positive vs. double positive, 4 (1–22) vs. 3 (1–5) vs. 4 (2–6), *P*=0.1296). The females represented a higher proportion of double-positive patients. A trend toward AQP4-ab positivity was present in patients who were older at disease onset than those in the other two groups (43 vs. 32 vs. 32, *P*=0.1925). The relapsing disease marked 100% of double-positive patients, 96% of AQP4-ab-positivepatients and 57% of MOG-ab-positive patients (*P*<0.0001).

**Figure 3:**
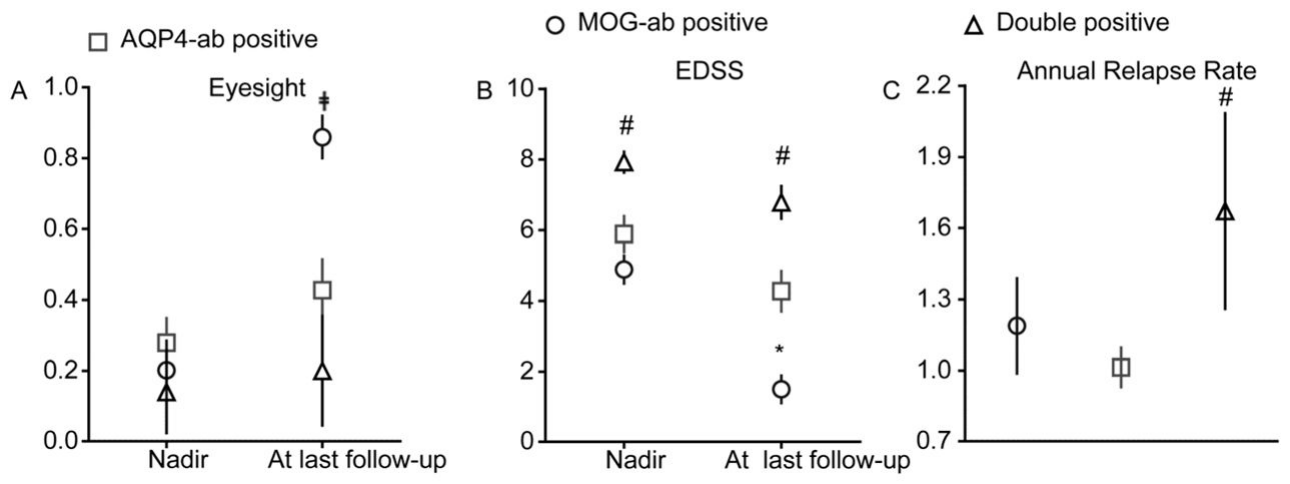
Clinical attacks, disability outcomes and relapse among NMOSD patients with different autoantibody types. A, Visual acuity. B, Expanded disability status scale (EDSS). C, Annual relapse rates.*, *P*<0.05, MOG-ab positive versus AQP4-ab positive. #, *P*<0.05, Double positive versus AQP4-ab positive. ╪, *P*<0.05, MOG-ab positive versus Double positive. Mean±SE is shown.

All patients in each group received corticosteroids during acute relapse, using a standard regime of intravenous methylprednisolone. There was no difference of visual acuity at nadir in the three groups. However, at the last follow-up, the visual acuity was significantly poorer in the double-positive group (Figure 3A), and these patients suffered the worst recovery from an acute episode, with little change in visual acuity. In the MOG-ab-positive patients, though, recovery of visual acuity was the best of all groups. The median change in visual acuity between episode nadir and the last follow-up was significantly less in double-positive patients as compared to the other two groups (double positive- vs. AQP4-ab-positive patients, 0.06 vs. 0.16, *P*=0.04447; AQP4-ab positive- vs. MOG-ab-positive patients, 0.16 vs. 0.53, *P*< 0.0001) (Figure 3A).

The mean of nadir EDSS was significantly different among the three groups (AQP4-ab-positive group vs. MOG-ab-positive group vs. double-positive group, 5.9±0.5 vs. 4.9±0.4 vs. 7.9±0.3, mean, SE; *P*<0.0001). Nadir EDSS of the double-positive group was significantly higher than for the AQP4-ab-positive group (*P*=0.0264), and nadir EDSS of MOG-ab-positive group was similar with AQP4-ab-positive group (*P*=0.2125). Mean disability at the last follow-up EDSS was 4.3±0.6 in the AQP4-ab-positive group, 1.5±0.4 in the MOG-ab-positive group, and 6.8±0.5 in the double-positive group. At that time, the EDSS of double-positive patients was higher than the AQP4-ab-positive ones (*P*=0.0161), and the last EDSS follow-up of the MOG-ab-positive group was lower than the AQP4-ab-positive group (*P*=0.0083). Recovery of double-positive patients from acute episodes, including little change in EDSS scores, was the worst of any group. In fact, the median change in EDSS scores between episode nadir and the present was significantly less in this group compared with other groups (double-positive vs. AQP4-ab-positive patients, 1.1 vs. 1.6, *P*=0.0484; AQP4-ab-positive vs. MOG-ab-positive patients, 1.6 vs. 3.4, *P*=0.0030) (Figure 3B).

All double-positive and AQP4-ab-positive patients and 60% of MOG-ab-positive patients were treated with an immunosuppressant after disease onset. Although a greater proportion of double-positive patients were treated with an immunosuppressant, their median annual relapse rate was significantly higher than in the other groups (AQP4-ab-positive vs. MOG-ab-positive vs. double-positive: 1.0±0.1 vs. 1.2±0.2 vs. 1.7±0.4, mean, SE; *P*=0.0431). Annual relapse rates of double-positive-patients were higher than for the AQP4-ab-positive patients (*P*=0.0196), but annual relapse rates of MOG-ab-positive patients did not differ notably from that of AQP4-ab-positive patients (*P*=0.0797) (Table 3, Figure 3C).

### Distribution of brain lesions varied among NMOSD patients with different autoantibodies

**Figure 4:**
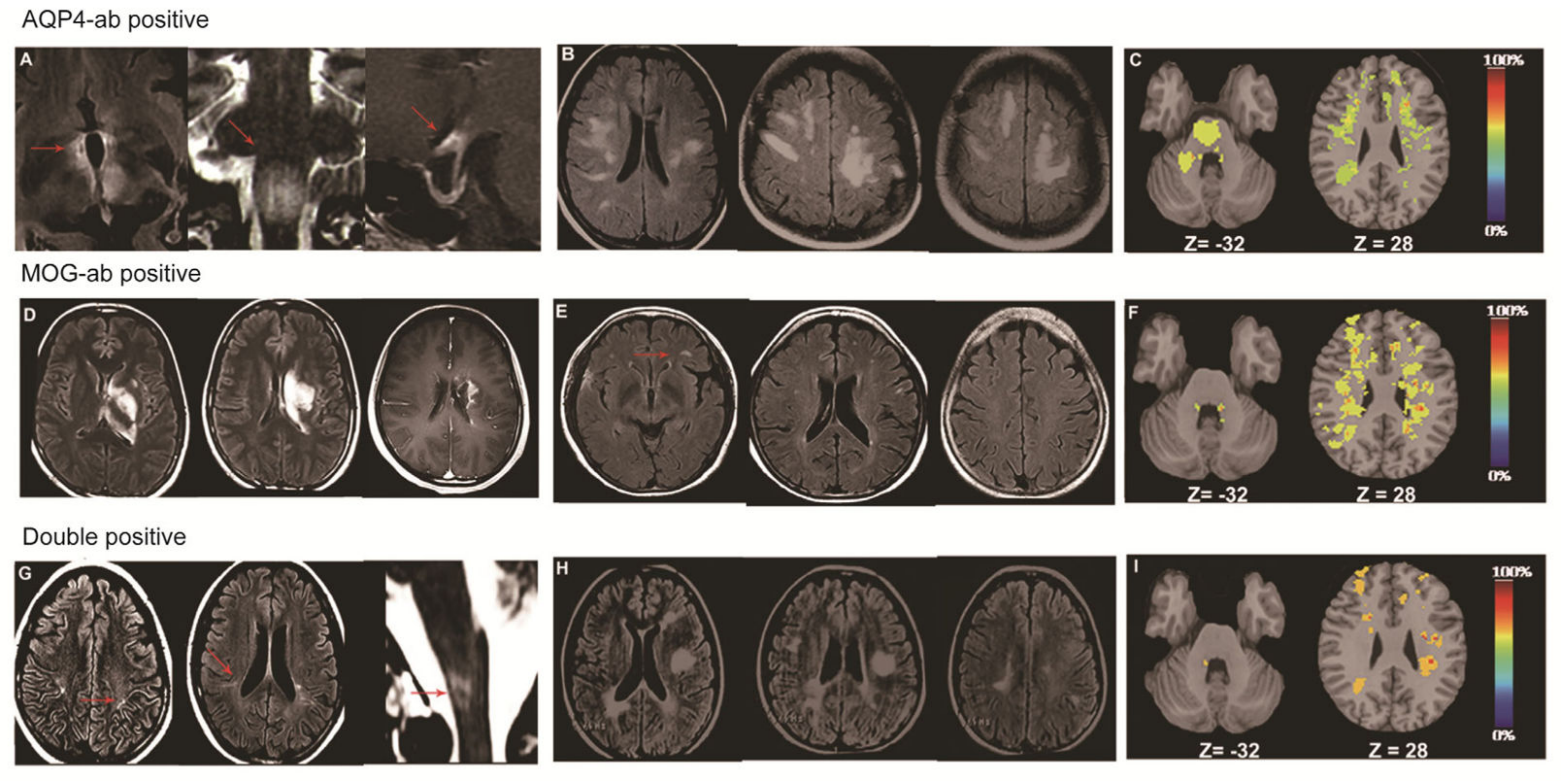
Brain lesions in MRIs of NMOSD patients with different antibody profiles during attacks. A–C, NMOSD patients with AQP4-ab. D–F, NMOSD patients with MOG-ab. G–I, NMOSD patients with both AQP4 and MOG-abs. A, Typical NMO-like lesion: fluid-attenuated inversion recovery sequence (FLAIR) with T2-weighted hyper-intensities evident in peri-ependymal areas and the diencephalon, including bilateral hypothalamic lesions (A left, arrows); T2-weighted hyperintensities in the floor of the fourth ventricle, bilateral area postrema lesion (A middle, arrow); chiasmal lesion with gadolinium enhancement (A right, arrow). B, ADEM-like lesion without enhancement: patient with extensive bihemispheric subcortical white matter FLAIR signals abnormality without enhancement. The lack of enhancement suggests an intact blood-brain barrier and supports a unique mechanism of edema induction from the dysfunction of water channels. C, Lesion probability distribution for NMOSD patients with AQP4-ab (*n*=26): the color scale (from 0% to 100%) represents the minimum to maximum probability of a lesion occurring in a particular spatial location. Montreal Neurological Institute (MNI) standard space template Z coordinate is shown in millimeters. D, AMED like lesions with enhancement: a patient with MOG-ab had a large confluent FLAIR signal abnormality in the left putaman, thalamus and paraventricle white matter that demonstrates diffuse gadolinium enhancement. Emerging enhancement suggests disruption of the blood-brain barrier and supports an inflammatory mechanism producing edema. E, MS-like lesion but lesions were too few to satisfy the Barkhof criteria for MS (arrow). F, Lesion probability distribution for NMOSD patients positive for MOG-ab (*n*=8). G, Small MS-like lesions satisfy Barkhof criteria, also a special NMO-like lesion with area postrema lesion surprisingly happened in the same patient (arrow). H, Large typical MS-like lesions satisfy Barkhof criteria. I, Lesion probability distribution for NMOSD patients with MOG-ab and AQP4-ab (*n*=10).

Ten (100%) double-positive patients, 26 (38%) AQP4-ab-positive patients and 8 (57%) MOG-ab-positive patients (all with the NMO phenotype) had brain lesions visible on MRI scans during the acute episode, but most brain lesions were asymptomatic (Table 3). Of the 26 AQP4-ab-positive patients with brain lesions, 17 (65%) had NMO-like brain lesions (i.e., periependymal areas, optic chiasm and area postrema) (Figure 4A), six (23%) patients had ADEM-like brain lesions, most of which were bilateral, asymmetric lesions in the supratentorial space without enhancements (Figure 4B), and three (12%) patients had nonspecific brain lesions. The lack of enhanced ADEM-like brain lesions in AQP4-ab-positive patients suggested an intact blood-brain-barrier and supported a unique mechanism of edema induction due to the dysfunction of water channels (Eichel et al., 2008).

Comparatively, eight MOG-ab-positive patients with brain lesions included five patients with MS-like lesions (i.e., periventricular, juxtacortical, temporal, occipital, and infratentorial), but those lesions were too few to satisfy MS criteria (Figure 4E). The other three patients had ADEM-like lesions, consisting of deep gray matter and fluffy white matter with enhancement (Figure 4D). The emergence of ADEM-like lesion enhancement suggested a disruption of the blood-brain barrier and supported an inflammatory mechanism that produced edema.

As to double-positive patients, seven of 10 exhibited MS-like brain lesions, and the other three had ADEM-like lesions. Of the seven double-positive patients, five had small MS-like brain lesions, and two had large confluent MS-like brain lesions (Figure 4G, H). Interestingly, one double-positive patient with intractable hiccough and nausea attacks had a small MS-like brain lesion that satisfies the Barkhof criteria and a confluent NMO-like lesion (involving postrema area) (Figure 4G). Three double-positive patients had deep gray matter and fluffy white matter lesions with or without enhancement, but did not present with fever, meningeal signs, or acute encephalopathy at the time of attack.

The quantitative probability analysis approach was adopted to document the brain lesion distribution among the three groups described here, with the respective results shown in Figure 4C, F and I. Distinguishing features of note were that more lesions in the medulla oblongata appeared in the AQP4-ab-positive group, whereas in the MOG-ab-positive group and double-positive group, more lesions were noted bordering the lateral aspect of the lateral ventricle’s body similar to that in MS. The majority of lesions in the AQP4-ab-positivepatient group were smaller in size and fewer in number compared with those in the double-positive and MOG-ab-positive groups.

### MOG-ab- and AQP4-ab-double positive patients have distinct spinal lesions

**Figure 5:**
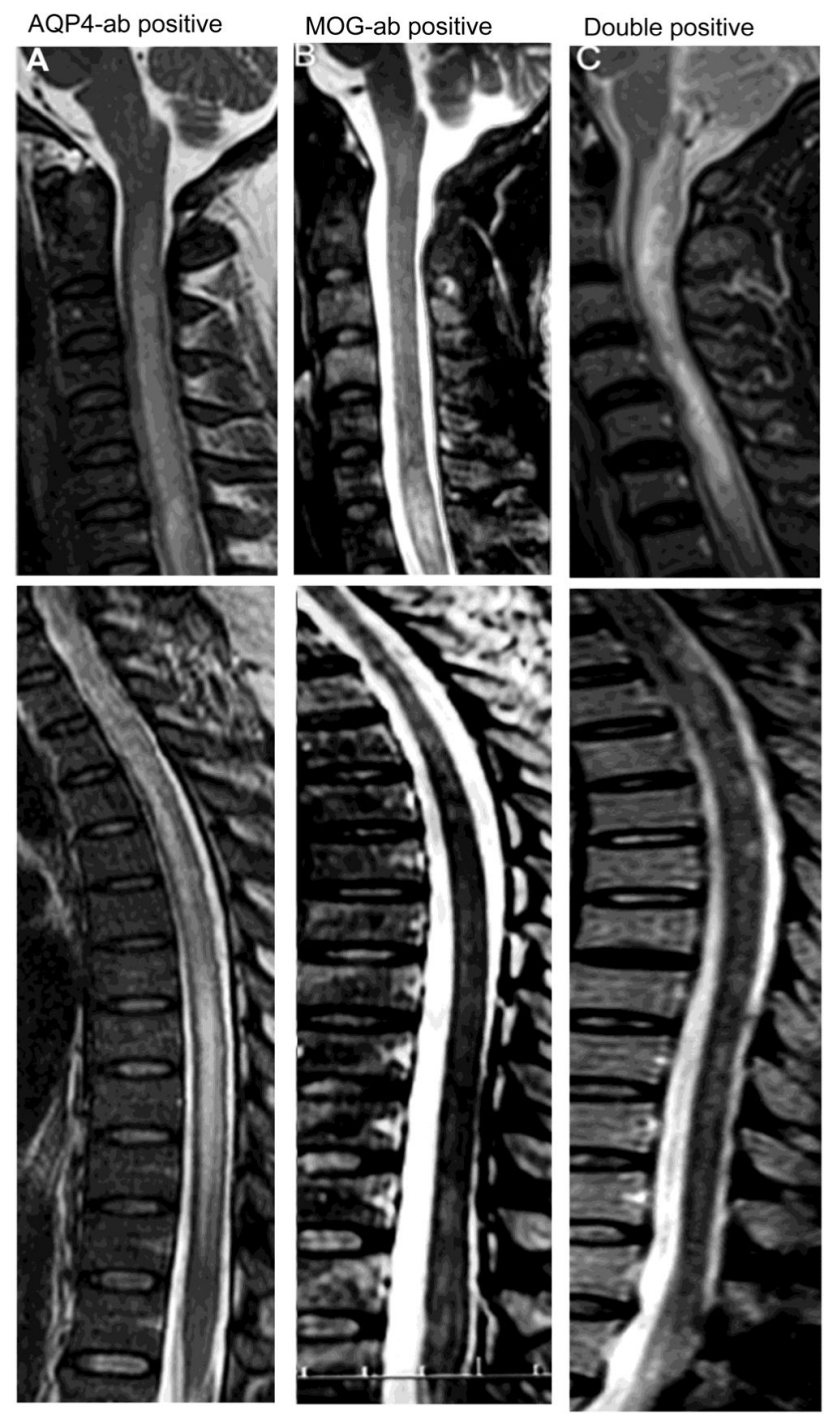
Spinal lesions in NMOSD patients with different antibody profiles during attacks. A, Sagittal T2 weighted images demonstrating single typical longitudinally extensive T2 hyperintense, central and severer edema spinal cord lesion from the cervical to thoracic cord of a patient with AQP4-ab. B, Sagittal T2 weighted images of multiple spinal lesions in a patient with MOG-ab: longitudinally extensive T2 hyperintensity and mildly edematous spinal cord lesion affect the cervical cord. Multiple discontinuous, short-segments, patchy signal abnormalities affect the thoracic cord to conus area. C, Sagittal T2 weighted images of multiple spinal lesions in a double-positive patient: longitudinally extensive T2 hyperintense, central and severe edematous spinal cord lesion affects the cervical cord and multiple discontinuous, short-segment, patchy signal abnormalities affect the thoracic cord to conus area.

We recorded that 61% of AQP4-ab-positive patients, 71% of MOG-ab-positive patients and 80% of double-positive patients had clinical evidence of spinal cord involvement at the first attack of NMOSD. The spinal cord MRI features of the patients are shown in Table 3. The conus was significantly more likely to be involved in the double-positive group and MOG-ab-positive patients (Figure 5). Among these three groups, we found significant differences as to the presence of cord edema and of multiple spinal cord lesions (Table 3, Figure 5).

### Severe atrophy of optic nerve and reduction of retinal nerve fiber layer thickness in double-positive patients

**Figure 6:**
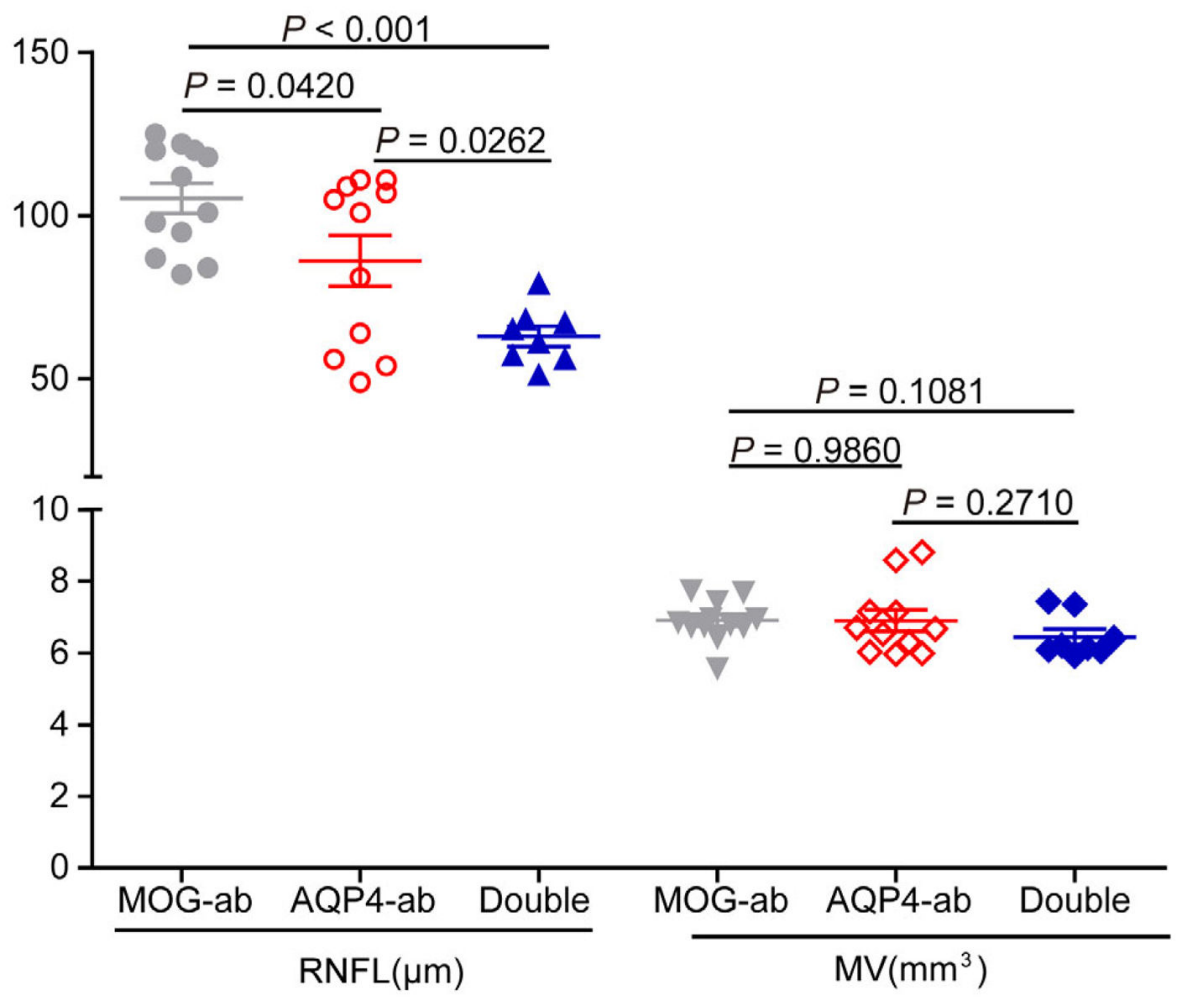
OCT measurements in patients with MOG-ab, AQP4-ab and double positive. ab, antibody. RNFL, retinal nerve fiber layer. MV, macular volume.

**Figure 7:**
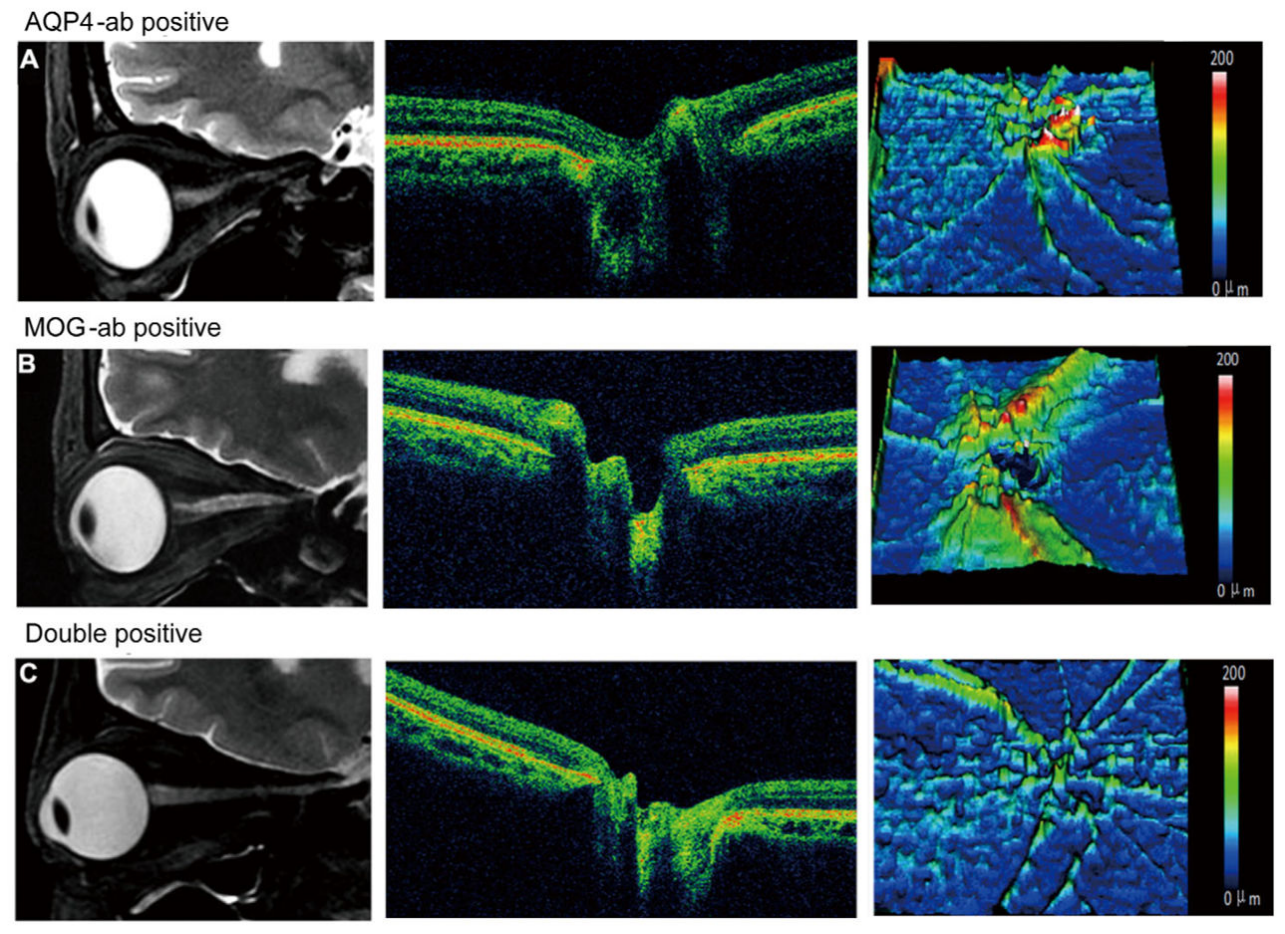
Optica nerve and retinal nerve fiber layer thickness in NMOSD patients with different antibody profiles at the last follow-up. A, Images from a patient with AQP4-ab and a 5-year course of disease. B, Images from a patient with MOG-ab and a 5-year course of disease. C, Images from a patient with both AQP4-ab and MOG-ab and a 2-year course of disease. Left, optic nerve MRI. Middle, Optical coherence tomography images of the peri-papillary area of retinal nerve fiber layer. Right, Thickness maps of the retinal ganglion cell layer of retinal nerve fiber. Optica nerve demyelination is evident as atrophy on optic nerve MRI, and neuroaxonal retinal damage appears as thinning of peri-papillary tissue measured by OCT. This condition was more severe in eyes of patients with optic neuritis who had AQP4-ab and MOG-ab than in those with either AQP4-ab alone or MOG-ab alone.

Optic nerve MRI and optical coherence tomography were more sensitive assessment for retinal nerve fiber layer (Liu et al., 2015) and performed in seven double-positive patients, six MOG-ab-positive patients and 23 AQP4-ab-positive patients during the post-testing recovery period. Compared to AQP4-ab-positive patients, optical coherence tomography imaging showed a decrease in retinal nerve fiber layer thickness in double-positive patients (63±3 vs. 86±8 μm, *P*=0.0262), an increase in MOG-ab-positive patients (102±11 vs. 86±8 μm, *P*=0.0420) (Figure 6) In MOG-ab-positive patients, only those whose eyes were obviously affected had slight optic nerve atrophy and attenuation of retinal nerve fiber layer thickness; unaffected eyes were completely normal. In contrast, the clinically unaffected eyes in two double positive patients had obvious optic nerve atrophy and retinal nerve fiber layer thickness attenuation. Eight AQP4-ab-positive patients also had marked optic nerve atrophy and attenuated thickness of the retinal nerve fiber layer in clinically unaffected eyes. Optic nerve atrophy in affected eyes of double-positive patients was the most severe among the three groups and was the least severe in MOG-ab-positive patients. Results for retinal nerve fiber layer thickness were similar; attenuation was worst in the double-positive patients but hardly deviated from normal in MOG-ab-positive patients (Figure 7).

## DISCUSSION

In this study, sera from 10 of 125 NMOSD patients tested positive for both AQP4 and MOG autoantibodies. Here, we noted that MOG-ab in NMOSD patients with or without AQP4-ab defined two phenotypes of the disease, each having distinctive clinical and radiologic (MRI) features. NMOSD patients carrying AQP4-ab and MOG-ab presented with a multiphase disease course, a heightened relapse rate and residual disability; that is, 70% of the double-positive patients had MS-like brain lesions. Spine lesions visible on MRI scans of double-positive patients more frequently had a multifocal distribution complicated by severe edema from the cervical to conus regions. Neurologic disability assessed by EDSS and visual acuity was more prominent in these patients as well. Retinal nerve fiber layer thickness attenuation in these patients were more severe than in their single-positive counterparts, worsening the former group’s visual function. One double-positive patient suffered the simultaneous occurrence of MS-like lesions, typical of those in the brains of MOG-ab-positive NMOSD individuals, along with an NMO-like lesion in the postrema area, typical of brain lesions of the AQP4-ab single-positive NMOSD group (Figure 4G). Similarly, the double-positive patients bore features of prototypic AQP4-ab and MOG-ab single positive patients, such as spinal cord lesions.

In the presence of AQP4 and MOG autoantibodies, NMOSD patients manifested the poorest visual acuity, least recovery from the acute visual episodes and higher EDSS scores than the AQP4-ab-positivepatients, indicating significantly more severe outcomes than patients with only MOG-ab or AQP4-ab.

We used FACS assays to test the serostatus of AQP4-ab and MOG-ab. Our results are contradictory to those in several recent reports, in which MOG-ab was found only in AQP4-ab seronegative NMOSD patients (Kitley et al., 2014; Kitley et al., 2012; Mader et al., 2011; Rostasy et al., 2013; Sato et al., 2014; Tanaka and Tanaka, 2014). However, others noted one double-positive patient among 20 with NMOSD (Kezuka et al., 2012). The cause of these controversial results may be the different detection methods used or a different part of antigen expressed on the cells selected as its carrier (Kitley et al., 2014; Weinshenker and Winger- chuk, 2014). Rostasy et al. and Mader et al. used 293T cells that transiently expressed the full-length MOG-EGFP fusion gene to test MOG-ab, but their cell surface staining technique would identify only antibodies binding to the extra-cellular domain of MOG. Kitley’s group published two papers on MOG-ab detection (Rostasy et al., 2013; Mader et al., 2011; Kitley et al., 2012, 2014), but they also used C-terminal-truncated MOG-EGFP fusion genes, which detect only antibodies on the cell surface. Tanaka and Sato used full-length MOG transiently transfected HEK 293 cells for MOG-ab detection (Sato et al., 2014; Tanaka and Tanaka, 2014), but Tanaka’s team transfected MOG only, and the Sato group transfected a MOG-IRES-DsRed cassette. Both of these constructs lack signals for the efficient monitoring of MOG expression. Moreover, the fluorescence microscope-based cell binding assay was applied for antibody detection in those reports that differ from ours, and the specificity and sensitivity may be different among methods (Reindl et al., 2013).

In agreement with the recent studies of cohorts from Oxford, England (Kitley et al., 2014), Japan and Brazil (Sato et al., 2014), analyses of our patient cohorts from Tianjin and Barrow revealed that some MOG-ab-positive patients presented with simultaneous optic neuritis as well as myelitis and appeared to have a monophasic disease course with relatively mild residual disability. The ADEM-like brain lesion and spinal lesion involving the conus more often appeared on MRI scans of patients who were MOG-ab positive. Importantly, comprehensive analysis of MRI and OCT parameters disclosed additional features in these MOG-ab-positive patients: multifocal lesions with mild edema visible on spinal MRI; greater prevalence of lateral ventricle lesions on brain MRI, mild optic nerve atrophy and nearly normal retinal nerve fiber layer thickness all contributing to residual but mild eyesight disability.

On the basis of these results, we propose that patients with NMO/NMOSD who carry MOG-ab, but not AQP4-ab, exhibit an intermediate disease phenotype of classical NMO and relapsing MS, which was consistent with Pröbstel’s group study (Marta et al., 2005). More importantly, we have identified for the first time, patients carrying antibodies to both AQP4 and MOG who exhibit the overlapping features of prototypic NMO and relapsing-form of MS.

Our observations that NMO/NMOSD associated with MOG-ab characterized cardinal clinical and MRI features that are independent and distinct from those of AQP4-ab (Kitley et al., 2014; Kitley et al., 2012; Mader et al., 2011; Rostasy et al., 2013; Sato et al., 2014; Tanaka and Tanaka, 2014) suggest two possibilities. First, MOG may serve as an autoantigen that, in conjunction with AQP4, acts synergistically to produce the immune-mediated tissue injury typically present in NMO and MS patients with both sets of these autoantigens. Second, MOG may also serve as a sole autoantigen, capable of initiating and driving the progression of NMO. MOG is a minor myelin antigen and constitutes only 0.05% of myelin proteins in the central nervous system (CNS) (Reindl et al., 2013). Nevertheless, several indicators hint that human MOG antibodies can induce cytotoxicity *in vitro* and *in vivo* (Mader et al., 2011; Saadoun et al., 2014). Furthermore, MOG-ab obtained from NMO patients and microinjected into mouse brains directly damaged myelin in a way that differed markedly from the effect of AQP4-ab and was reversible (Bettelli et al., 2006). In addition, MOG T cell-receptor and B cell-receptor do spontaneously developed NMO-like optic nerve and spinal cord lesions suggesting that MOG-specific immune responses might initiate demyelinating diseases in the CNS (Berger et al., 2003).

A question remains as to whether MOG-ab in NMOSD patients is derived from secondary demyelination that subsequently elicits an autoantibody response. The fact that the disease duration in MOG-ab-positive patients was three (1–5) years and four (1–22) years in AQP4-ab patients argues against this possibility. Most importantly, since 75% of seronegative patients developed MOG-ab within four months of neurological disease onset in Tanaka’s study (Tanaka and Tanaka, 2014), and 100% of AQP4-ab sero-negative patients developed MOG-ab at the time of disease onset in Kitley’s study (Kitley et al., 2014), the suggestion that MOG-Ab in NMO does not emerge from secondary demyelination seems logical.

Our study has several limitations. First, since double-positive patients appear to be rare (10/125), the unique features described in this study await further verification. Second, no longitudinal monitoring was done of MOG-ab titers relative to disease manifestation. Third, regardless of numerous attempts to establish a role for MOG-ab in patients with MS, the results have been controversial (Kuhle et al., 2007; Probstel et al., 2015). Therefore, the worth of identifying MOG-ab in NMOSD must be evaluated prospectively in multi-center studies that use identical detection methods. Despite these limitations, our study may be instructive in managing these patients.

Specifically, presence of MOG-ab with or without AQP4-ab may assist to predict a clinical course of a given patients, i.e. prototypic NMO, combined or intermediate between MS and NMO. The fact that three of our 10 double-positive patients did not respond to rituximab or several other immunologic therapies (Table 2) raises the question whether we should treat these patients more aggressively to halt the disease progression. Some patients with MOG-ab might represent an intermediate phenotype between the markedly different NMOSD and MS, whose crossover disease was diagnosed at different stages of each. If so, the discrepancies in results from MOG-ab assays relative to MS might be explained (Kuhle et al., 2007; Probstel et al., 2015). How to diagnose and treat such patients is a new challenge, the first steps of which might stem from our detailed results.

## MATERIALS AND METHODS

### Patients

Subjects included in this study were adult NMOSD patients seen in the Department of Neurology, Tianjin Medical University General Hospital, Tianjin, China and in the Barrow Neurological Institute, Phoenix, AZ, USA. All these patients met the diagnostic criteria for NMOSD proposed in 2007 by Wingerchuk et al., including patients with either optic neuritis (ON) with AQP4 antibody or longitudinally extensive transverse myelitis (LETM) which experienced of LETM presented T2 hyperintensity on spinal cord MRI extending over ≥3 vertebral segments, without optic neuritis, not satisfy diagnostic criteria for MS and a full autoimmune, infection, tumor, paraneoplastic syndrome and metabolic screen, no identifiable causes of LETM (Wingerchuk et al., 2007); also included were those who met the McDonald Criteria for multiple sclerosis (MS) as revised in 2010 (Figure 1) (Polman et al., 2011). Collected serum samples were stored at −80°C until analysis. Sera from 45 patients with miscellaneous neurological diseases (acute cerebral stroke 17, epilepsy 10, encephalitis five, Myasthenia gravis five, Guillain-Barre syndrome three, paraneoplastic syndrome five) and 45 healthy donors were stored in the same fashion and also included in this study (Figure 1). This is a retrospective clinical pilot study. From April to December 2013, patients with NMOSD seen during the routine follow-up at the both sites were consecutively enrolled and patient serum samples were routinely collected. AQP4-ab detection was performed at the time of enrollment. MOG-ab was examined afterward, i.e. whenever samples were available. Clinical and imaging data about the patients were respectively collected and analyzed. Written informed consent was obtained from all subjects before blood draws. The study was approved by the ethics committees of Tianjin Medical University and Barrow Neurological Institute.

### AQP4- and MOG-ab assays

Serum antibody detection was pursued at both institutional sites (Tianjin Medical University and Barrow Neurological Institute). Stable HEK-293T cell lines that express AQP4 M23-EGFP or MOG alpha 1-EGFP fused genes were used as antibody harboring cells. EGFP was fused to the C-terminal of AQP4 or MOG gene that linked with a flexible amino acid sequence “GGGGS”. In-house FACS assay was used to detect antibody binding of patient serum IgG to surface AQP4 or MOG transduced in HEK293T cells. The antibody binding detection was scored by two investigators independently. For autoantibody detection, AQP4-EGFP/ 293T cells were incubated with diluted human sera (1/10) and MOG-EGFP/293T cells were incubated with diluted human sera (1/128) in FACS buffer (PBS containing 1% BSA and 0.03% NaN3) for half hour, EGFP/293T cells were used as control. After washing, the cells were incubated with PerCP-Cy5.5-conjugated goat anti-human IgG Ab (Biolegend, America). Half hour later, the cells were washed once with FACS buffer and then analyzed on a FACS Calibur (BD Biosciences), and PerCP-Cy5.5 mean fluorescence intensity (MFI) was measured on EGFP positive cells (Figure 2). (MFI AQP4- or MOG-EGFP-transfected cells/MFI EGFP-transfected cells) were reported as AQP4 or MOG-IgG Binding Index; values of ≥3 were considered positive.

The FACS-based cell-binding assays for the MOG- antibodies were validated using 90 controls (45 healthy volunteers, and 45 patients with miscellaneous neurological diseases (acute cerebral stroke 17, epilepsy 10, encephalitis five, Myasthenia gravis five, Guillain-Barre syndrome three, and paraneoplastic syndrome five)). None of the control was positive for MOG-antibodies using our full-length MOG-assay with titers ≥1:128 dilution. Considering MOG-antibodies have been reported to be clinically relevant at high titers, we have used the 1:128× dilution for the current study.

### Clinical and imaging studies

The demographic and clinical data of individual participants were recorded and analyzed. The expanded disability status scale (EDSS) scoring system and visual acuity were used to estimate disability during and after each episode of disease onset. The nadir EDSS score was taken as the maximum EDSS score, and the nadir visual acuity was taken as the minimum register value during each acute episode. Visual acuity was measured by the standard logarithmic acuity chart with decimals register. Visual acuity less than 0.02 were registered as zero. Severe visual disability was defined as visual acuity of 0.1 or less in one or both eyes at last follow-up.

The visual appearance in MRI scans of spinal cord and brain at episodes of relapse and subsequent clinical recovery was reviewed against a pro forma by a neuroradiologist experienced in appraising inflammatory disorders and, for this project, blinded to clinical features and antibody status. Brain MRI scans were classified as normal, nonspecific, MS- like, NMO-like, or acute disseminated encephalomyelitis (ADEM)-like lesions in several specific locations. Scans were classified as MS-like if lesions were seen in regions considered typical of MS (i.e., periventricular, juxtacortical, temporal, occipital, and infratentorial); the term “nonspecific” described scans with a small number of white matter lesions with no MS features (Kitley et al., 2014). Lesions were classified as NMO-like when surrounding the fourth ventricle, hypothalamus or aqueduct lesions as previously described (Pittock et al., 2006; Tackley et al., 2014), and scans with lesions in deep gray nuclei or fluffy white matter were classified as ADEM-like (Dale et al., 2000; Kitley et al., 2014). Spinal cord MRI scans were evaluated for lesion length, axial and sagittal location, and the presence of cord edema.

A quantitative probability analysis approach was used to document the distribution of brain lesions in a cohort of AQP4-ab-positive patients, MOG-ab-positive patients and double-positive patients, which would be particularly relevant to patients who present with a spatially limited phenotype. MRI data were analyzed using the FMRIB Software Library of tools (University of Oxford, UK). For each subject, hyperintense T2 lesions were marked and segmented manually on an axial FLAIR image, in native space, with simultaneous reference to the T2 scan. The FLAIR images were then registered in the Montreal Neurological Institute (MNI) 2-mm standard space template using a nonlinear transformation method (FNIRT (FMRIB’s nonlinear image registration tool)). The nonlinear transformation matrix was then applied to the respective lesion segmentation masks to transform them into the space of the standard template. After transformation, the lesion masks were thresholded at 50% and binarized again to avoid the volume increase caused by the trilinear interpolation. They were then summed and averaged for each subject group to create lesion probability maps (Matthews et al., 2013).

### Optical coherence tomography

SD-OCT (3D OCT-2000; Topcon Corp, Tokyo, Japan) was used to obtain tomographic images of the parapapillary fundus with the three-dimensional (3D) disc scan and 3D Macula scan (128 horizontal scan lines comprised of 512 A scans for an image area of 6 mm×6 mm). OCT scanning was performed by a trained technician who monitored scans to ensure reliable fixation. Scans with a signal strength of 7/10 or with artifact were excluded from analysis. Macular cube and retinal nerve fiber layer thickness scans were further analyzed in a blinded fashion.

### Statistical analysis

SPSS for Windows version 17.0 software (SPSS, Inc, Chicago, IL, USA) was used for the analyses. Continuous variables are reported as mean±SE or medians (range), and categorical variables appear as percentages. Continuous variables comparison among groups by means of the one-way ANOVA test followed post-hoc testing, or Mann-Whitney test and Chi-square test or Fisher exact testing for qualitative data. Statistical significance is defined as *P*<0.05.

## Figures and Tables

**Table 1:** Treatment status at time of blood sampling of patients with NMOSD

Drugs	Number of patients	Treat time before sampling, median (range), month
No treatment	12	8 (3–48)
Rituximab	5	24 (18–28)
Azathioprine	33	20 (3–60)
Oral corticosteroids	50	18 (2–35)
Azathioprine, oral corticosteroids,	11	13 (4–38)
Hydroxychloroquine, Methotrexate	1	3
Cyclophosphamide	7	15 (8–26)
Methotrexate	5	11 (6–24)
bimonthly, intravenous immunoglobulin	1	12

**Table 2:** Clinical characteristics of ten NMOSD patients with MOG antibody and AQP4 antibodya)

Patient	Onset age, year/sex	Disease Duration, year	No. of attacks	Phenotype	First attack site/EDSS	Severe visual disability	BS symptoms	EDSS at last flow-up	Brain MRI	Spinal MRI lesion	CSF OCB	Other serumauto-antibodies	Treatment
1	15/F	6	3	NMO	LETM+ON+Brain/7	Yes	No	6	Large MS-like lesions	C_4_-Conus	No	No	CS, AZA
2	36/F	6	8	LETM	LETM/8	No	No	7	Small MS-like lesions	T_1_-Conus	No	No	RTX
3	18/F	2	8	NMO	LETM+ON+Brain/8.5	Yes	Yes	8	Small lesions	MS-likeC_1_-Conus	No	Yes	Met, HCQ
4	60/F	4	6	NMO	LETM/8.5	Yes	No	8.5	Small MS-like lesions	C_2_-Conus	No	No	CS, AZA
5	49/F	2	3	NMO	LETM/7.5	Yes	No	5	Small MS-like lesion	C_1_-Conus	No	No	CS, AZA
6	33/F	4	6	NMO	ON+Brain/3	No	No	6	Large MS-like lesions	C_3_-Conus	Yes	No	CS, AZA
7	15/F	5	7	NMO	ON/1	Yes	No	9	ADEM-like lesions with enhancement	C_1_-Conus	No	No	CS, AZA
8	20/F	5	10	NMO	LETM+ON/6.5	Yes	No	9	ADEM-like lesions without enhancement	C_1_-Conus	No	No	CS, AZA
9	30/F	3	4	NMO	LETM/7.5	No	No	8.5	ADEM-like lesions without enhancement	C_1_-Conus	No	No	RTX
10	36/F	3	6	NMO	LETM/8	Yes	No	6	Small MS-like lesions	C_1_-T12	No	No	RTX

a) RTX, Rituximab. IVIG, intravenous immunoglobulin. HCQ, Hydroxychloroquine. Met, Methotrexate. AZA, azathioprine. BS, brainstem. CS, oral corticosteroids. EDSS, Expanded disability status scale score. LETM, longitudinally extensive myelitis. NMO, neuromyelitis optica. NMOSD, neuromyelitis optica spectrum disorders. OCB, oligoclonal bands. ON, optic neuritis. MS, multiple sclerosis. ADEM, acute disseminated encephalomyelitis. Severe visual disability was defined as visual acuity of 0.1 or less in one or both eyes at last follow-up. Other serum autoantibodies include antinuclear antibodies, anti-Sjögren’s syndrome A antibodies, anti-Sjögren’s syndrome B antibodies, thyroglobulin antibodies, and thyroid peroxidase antibodies.

**Table 3:** Comparison of clinical features, MRI and laboratory findings between NMOSD patients with MOG antibody, AQP4 antibody and those seropositive, with statistical analysis among groupsa)

Clinical	AQP4-ab positive (*N*=69)	MOG-ab positive (*N*=14)	Double positive (*N*=10)	*P* value
NMO/ NMOSD-LETM/ NMOSD-ON, *n*	57/8/4	8/6/0	9/ 1/ 0	0.0438
Female sex, *n* (%)	56 (81%)	10 (71%)	10 (100%)	0.1975
Age at first attack, median (range), years	43 (20–63)	32 (15–56)	32 (15–60)	0.1925
Disease duration (time from disease onset to serum drawl), median (range), years	4 (1–22)	3 (1–5)	4 (2–6)	0.1296
Patients with a single attack, *n* (%)	3 (4%)	6 (43%)	0 (0%)	<0.0001
Simultaneous ON+myelitis attacks (any time)	17 (24%)	6 (43%)	3 (30%)	0.3788
Number of attacks, median (range)	3 (1–17)	2 (1–6)	6 (3–10)	0.0697
Brain MRI at attack				
Normal, *n*(%)	43 (62%)	6 (43%)	0 (0%)	0.0008
MS-like (lesion were too few to satisfy the Barkhof criteria for MS), *n*(%)	0(0%)	5 (36%)	0 (0%)	<0.0001
MS-like (satisfy Barkhof criteria), *n* (%)	0 (0%)	0 (0%)	7 (70%)	<0.0001
NMO-like, *n* (%)	17 (25%)	0 (0%)	0 (0%)	0.0268
ADEM-like with lesion enhancement, *n* (%)	0 (0%)	3 (21%)	1 (10%)	0.0010
ADEM-like without lesion enhancement, *n* (%)	6 (9%)	0 (0%)	2 (20%)	0.2264
Nonspecific, *n* (%)	3 (4%)	0 (0%)	0 (0%)	0.5833
Spinal MRI at attack				
Multiple spinal cord lesions, *n* (%)	0 (0%)	14 (100%)	10(100%)	<0.0001
Moderate cord edema, *n* (%)	0 (0%)	14 (100%)	0 (0%)	<0.0001
Serious cord edema, *n* (%)	65 (94%)	0 (0%)	10(100%)	<0.0001
CSF analysis at attack				
Cell count, median (range)	16 (0–276)	4 (0–14)	17 (0–128)	0.5165
Protein, median (range) mg/dl	39 (20–89)	50 (29–86)	44 (29–45)	0.3504
Oligoclonal bands positivity, *n* (%)	4(6%)	3 (21%)	1 (10%)	0.1616
Other serum autoantibodies	13 (19%)	2 (14%)	1 (10%)	0.5778

a) MRI, magnetic resonance imaging. NMOSD, neuromyelitis optica spectrum disorders. MOG, myelin oligodendrocyte glycoprotein. AQP4, aquaporin-4. ab, antibody. CSF, cerebrospinal fluid. Other serum autoantibodies include antinuclear antibodies, anti-Sjögren’s syndrome A antibodies, anti-Sjögren’s syndrome B antibodies, thyroglobulin antibodies, and thyroid peroxidase antibodies.
